# Systemic Immunotherapy for Urothelial Cancer: Current Trends and Future Directions

**DOI:** 10.3390/cancers9020015

**Published:** 2017-01-27

**Authors:** Shilpa Gupta, David Gill, Austin Poole, Neeraj Agarwal

**Affiliations:** 1 Masonic Cancer Center, University of Minnesota, Minneapolis, MN 55455, USA; guptash@umn.edu; 2 Huntsman Cancer Institute, University of Utah, Salt Lake City, UT 84112, USA; David.Gill@hsc.utah.edu (D.G.); Austin.Poole@hci.utah.edu (A.P.)

**Keywords:** urothelial carcinoma, bladder cancer, immunotherapy

## Abstract

Urothelial cancer of the bladder, renal pelvis, ureter, and other urinary organs is the fifth most common cancer in the United States, and systemic platinum-based chemotherapy remains the standard of care for first-line treatment of advanced/metastatic urothelial carcinoma (UC). Until recently, there were very limited options for patients who are refractory to chemotherapy, or do not tolerate chemotherapy due to toxicities and overall outcomes have remained very poor. While the role of immunotherapy was first established in non-muscle invasive bladder cancer in the 1970s, no systemic immunotherapy was approved for advanced disease until the recent approval of a programmed death ligand-1 (PD-L1) inhibitor, atezolizumab, in patients with advanced/metastatic UC who have progressed on platinum-containing regimens. This represents a significant milestone in this disease after a void of over 30 years. In addition to atezolizumab, a variety of checkpoint inhibitors have shown a significant activity in advanced/metastatic urothelial carcinoma and are expected to gain Food and Drug Administration (FDA) approval in the near future. The introduction of novel immunotherapy agents has led to rapid changes in the field of urothelial carcinoma. Numerous checkpoint inhibitors are being tested alone or in combination in the first and subsequent-line therapies of metastatic disease, as well as neoadjuvant and adjuvant settings. They are also being studied in combination with radiation therapy and for non-muscle invasive bladder cancer refractory to BCG. Furthermore, immunotherapy is being utilized for those ineligible for first-line platinum-based chemotherapy. This review outlines the novel immunotherapy agents which have either been approved, or are currently being investigated in clinical trials in UC.

## 1. Introduction

Urothelial cancer (UC) of the bladder, renal pelvis, ureter, and other urinary organs is the fifth most common cancer in the United States with an estimated incidence of 143,190 new cases in 2016. Of these, an estimated 76,960 cases are urothelial cancer of the bladder with 16,390 deaths expected in 2016 [1]. Bladder cancer-related mortality had remained unchanged for the last 30 years and platinum-based chemotherapy regimens remain the standard of care for first-line treatment of advanced/metastatic UC and the five-year survival rate is around 15% [2,3].

Until recently, there were no standard salvage systemic therapies for advanced/metastatic UC, and chemotherapy regimens were associated with modest survival advantage, and significant toxicities. In a remarkable development, in May 2016, the Food and Drug Administration (FDA) approved atezolizumab, a programmed death ligand 1 (PD-L1) inhibitor, for use in advanced UC patients who have progressed on a platinum-based regimen [4,5].

**Figure 1:**
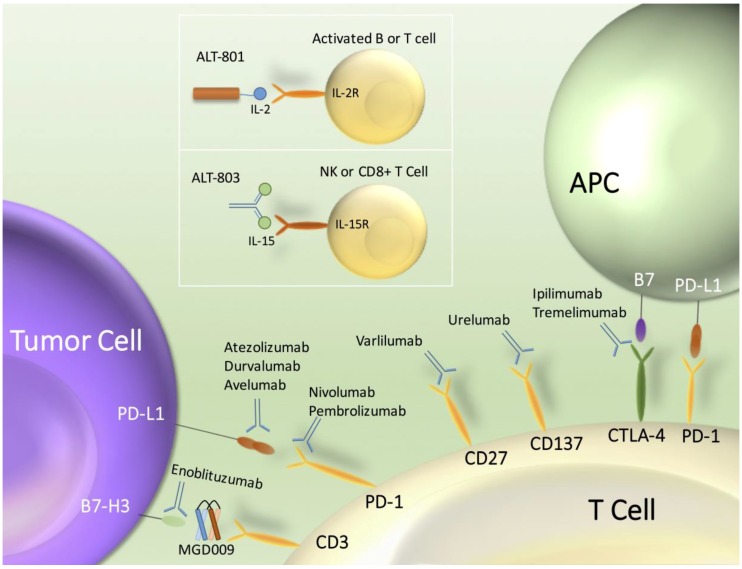
Checkpoint inhibitors, interleukin stimulators and novel immunotherapy agents in urothelial carcinoma.

Other checkpoint inhibitors investigated against UC include the monoclonal antibodies ipilimumab and tremelimumab which target cytotoxic T-lymphocyte-associated protein 4 (CTLA-4) and pembrolizumab, nivolumab, durvalumab, and avelumab targeting programmed cell death protein 1 (PD-1) or its ligand PD-L1. Other novel immunotherapy agents such as ALT-801, a tumor-targeted IL-2, and ALT-803, an IL-15 superagonist complex, have also shown activity in UC (Figure 1).

Atezolizumab, durvalumab and avelumab target PD-L1 on both the tumor and T cell. Nivolumab and pembrolizumab inhibits PD-1 on the T cell surface. Ipilimumab and tremelimumab inhibits CTLA-4 on T cells. ALT-801 and ALT-803 target and subsequently activate interleukin-2 (IL-2) and interleukin-15 (IL-15), respectively. B7-H3 is an immune checkpoint molecule expressed on tumors. CD27 and CD137 are co-stimulatory immune checkpoint molecules on T cells. Programmed death protein 1 (PD-1) is an immune checkpoint cell surface receptor on T cells and binds to it’s ligand, programmed death-ligand 1 (PD-L1) on both tumor cells and antigen presenting cells (APC). Cytotoxic T-lymphocyte-associated protein 4 (CTLA-4) is an immune checkpoint receptor expressed on T cells. MGD009 is a dual affinity re-targeting protein against B7-H3 and CD3. Enoblituzumab is a humanized IgG1 monoclonal antibody that binds to B7-H3. Varliumab is a monoclonal antibody against CD-27. Urelumab is a fully human monoclonal IgG4k antibody agonist against CD137, a tumor necrosis factor (TNF) family receptor expressed primarily on activated T cells and activated natural killer (NK) cells.

After the long void of no advances for advanced/metastatic UC, immunotherapy is changing the treatment paradigm for UC. Several novel immunotherapy agents, with unique mechanisms of action are currently being explored with or without checkpoint inhibitors in UC. Immunotherapies are being studied in first-line metastatic UC, non-invasive bladder cancer, neoadjuvant and adjuvant settings, as well as concurrently with radiation. (Table 1, Table 2 and Table 3) The role of checkpoint inhibitors and other novel immunotherapy agents, which have reached the advanced stages of development in UC, will be reviewed in the following sections.

## 2. Immunotherapy in Metastatic Refractory Urothelial Carcinoma

### 2.1. Checkpoint Inhibitors

#### 2.1.1. Atezolizumab

Atezolizumab (MPDL3280A) is an immunoglobulin G1 (IgG) monoclonal antibody directed against PD-L1 that initially showed promising activity against advanced/metastatic UC [4]. It received accelerated FDA approval in May 2016 for treatment of patients with advanced or metastatic urothelial carcinoma who have progressed after platinum-based therapy or who have progressed within a year of neoadjuvant or adjuvant treatment with a platinum-containing regimen [5]. Approval was based on a single-arm, multinational phase II study (IMvigor 210) with 315 patients which showed significant objective response rate (ORR) and durablility of responses [5].

In this study, in addition to having locally advanced or metastatic urothelial carcinoma, patients were required to have an inoperable disease with prior platinum-based therapy, with Eastern Cooperative Oncology Group (ECOG) performance status of 0 or 1, measurable disease defined by Response Evaluation Criteria In Solid Tumors version 1.1 (RECIST v1.1), adequate hematological and end-organ function, and no autoimmune disease or active infections. Seventy-three percent of patients had previously received cisplatin-based therapy and 26% had received carboplatin. Eighteen percent of patients had their first progression on the prior treatment regimen within 12 months: 41% had received two or more systemic regimens in the metastatic setting, and 24% of patients had received intravesical BCG (Bacillus Calmette-Guerin) therapy.

Patients received intravenous atezolizumab at a dose of 1200 mg, given every three weeks [5]. The primary outcome of ORR was obtained in 15% (45/310) of patients with 5% obtaining a complete response (CR). Formalin-fixed or fresh tumor tissues were obtained in all patients for PD-L1 staining; the PD-L1 expression status on infiltrating immune cells (ICs) in the tumor microenvironment was defined by the percentage of PD-L1 positive immune cells: IC0 (<1%), IC1 (≥1% but <5%), and IC2/3 (≥5%). Patients in the IC2/3 cohort demonstrated an ORR of 26% (26/100) with 11% obtaining a CR; an ORR and CR of 8% (8/103) and 2% (2/103) was seen in the IC0 cohort and 10% (11/107) and 2% (2/107) in the IC1 cohort.

While increased PD-L1 expression on immune cells correlated with an increased response, even patients with low or no PD-L1 expression seemed to derive benefit which, when compared to traditional salvage chemotherapy, was better in efficacy and tolerability. The presence of PD-L1 is not required for the current FDA approval. Presence of liver metastases correlated with decreased ORR (5% vs. 19%); presence of visceral metastases (ORR 10% vs. 31%) and ECOG of 1 compared to 0 (ORR 8% vs. 25%). With a median follow-up of 11.7 months, ongoing responses were seen in 38 (84%) of 45 responders. At the time of data cut-off, the median duration of response (DOR) had not been reached. Atezolizumab was very well tolerated, grade 3–4 treatment-related adverse events (TRAEs), of which fatigue was the most common, occurred in 50 (16%) of 310 treated patients. Grade 3–4 immune-mediated adverse events occurred in 15 (5%) of 310 treated patients, with pneumonitis, increased aspartate aminotransferase, increased alanine aminotransferase, rash, and dyspnea being the most common. No treatment-related deaths occurred during the study [5].

#### 2.1.2. Durvalumab

Durvalumab (MEDI4736) is a human IgG1 k monoclonal antibody targeting PD-L1. A phase I/II dose escalation and dose-expansion study evaluated its safety and efficacy in patients with advanced solid tumors [6]. The safety and efficacy of durvalumab was reported for the expansion cohort of patients with metastatic UC of the bladder [7]. This study was conducted at 70 centers worldwide and included patients with inoperable or metastatic transitional-cell UC who had progressed on, been ineligible for, or refused standard therapies. Inclusion criteria included ECOG 0-1, sufficient organ function, and PD-L1 tumor staining on fresh tumor biopsy or archived tissues. Patients were excluded for active autoimmune disease, prior severe or persistent immune-related adverse events (irAEs), prior immunotherapy directed against PD-1 or PD-L1, requirement of prednisone 10 mg daily or greater, and untreated brain metastases. Durvalumab was administered intravenously at 10 mg/kg every two weeks for 12 months or until disease progression, unacceptable toxicity, or study drug discontinuation for any other reason. Patients with disease progression were allowed to continue durvalumab if they were deriving clinical benefit. Additionally, patients could be retreated with a 12-month durvalumab course if they did not receive a different treatment and had not met criteria for discontinuation. Initially, 20 patients were enrolled regardless of PD-L1 expression in archival or fresh tumor biopsy tissue, but subsequent patients were required to have a minimum expression of 5% in tumor cells. After analysis of PD-L1 expression in the first 20 patients, a cutoff of 25% PD-L1 positivity was used for tumor cells or immune cells for response analysis [7].

The primary endpoint was safety and secondary endpoints were ORR (defined as CR, or partial response (PR)) and disease control rate at 12 weeks (defined as confirmed CR or PR, or stable disease). Exploratory analysis included assessment of PD-L1. Sixty-one patients were enrolled, 40 of whom were PD-L1 positive; all 61 patients were included in safety analysis, but only 42 were evaluable for response. Twenty-eight of 42 (66.7%) had PD-L1 expression on either tumor cells or immune cells. A majority of patients (93.4%) had received at least one line of therapy with 31.1% of patients receiving at least three previous lines of therapy. Liver metastases were present in 29.5% of patients and 23% of patients had a hemoglobin less than 10 g/dl, both known to be adverse prognostic risk factors.

Thirty-nine patients (63.9%) experienced TRAEs, with fatigue, diarrhea, and decreased appetite seen most frequently. A majority of TRAEs were low grade and grade 3 TRAEs occurred in three patients (4.9%). TRAEs of special interest were reported in 14/61 patients (23%). Most common were diarrhea, pruritus and infusion-related reactions. The ORR was 31%; 46.4% in the PD-L1 positive subgroup and 0% in the PD-L1 negative subgroup. The disease control rate, which includes those with ORR and stable disease at 12 weeks, was 57.1% in the PD-L1 positive subgroup and 28.6% in the PD-L1 negative subgroup. ORR was also analyzed based on PD-L1 expression on tumor cells or immune cells; it was reported to be 46.7% in the tumor cells PD-L1 positive subgroup vs. 22.2% in PD-L1 negative subgroup. Based on PD-L1 expression in the immune cells subgroup, disease control rate was 55.6% in the PD-L1 positive subgroup and 12.5% in the PD-L1 negative subgroup. Median follow-up was 6.5 months in response-evaluable patients with a median time to response of 6.3 weeks. Median duration of response had not been reached. In addition, 12/13 patients (92.3%) had an ongoing response at last follow-up [7].

Based on these promising results, durvalumab was granted FDA breakthrough therapy designation for patients with PD-L1 positive patients with inoperable or metastatic UC that had previously progressed on a standard platinum-based regimen. Durvalumab is being studied in several trials in UC, alone or in combination with tremelimumab (Table 1).

#### 2.1.3. Pembrolizumab

Pembrolizumab is a humanized monoclonal IgG4 antibody against PD-1, currently approved for the treatment of advanced melanoma, PD-L1-positive non-small cell lung cancer and for recurrent or metastatic head and neck squamous cell carcinoma with disease progression on or after platinum-containing chemotherapy [8]. The KEYNOTE-012 study with pembrolizumab was designed to investigate the safety, tolerability, and anti-tumor activity of pembrolizumab in patients with advanced gastric cancer, triple negative breast cancer, head and neck cancer, and UC. The safety and efficacy data for the UC cohort has been previously presented [9,10]. Patients with histologically or cytologically confirmed diagnosis of locally advanced or metastatic UC, including cancers of the renal pelvis, ureter, bladder, or urethra and both transitional and non-transitional histologies. There was no limit to the number of previous therapies. Patients were required to have measurable disease based on RECIST v1.1, ECOG performance status of 0–1, adequate organ function, and presence of at least 1% PD-L1 expression detected on the tumor or in tumor stroma in fresh biopsy or archival tissues. Key exclusion criteria included active autoimmune disease, interstitial lung disease, additional malignancy, central nervous system metastases, and active infections. Patients who had received prior treatment with T-cell co-stimulation or checkpoint pathways were excluded. Patients were treated with pembrolizumab 10 mg/kg every two weeks until CR, progression, or unacceptable toxicity. Thirty-three patients were enrolled, 33% of patients had received ≥3 prior therapies and 66% of patients had visceral or osseous metastases. The primary endpoints were safety, tolerability and ORR assessed per RECIST v1.1 by central imaging vendor review. Secondary endpoints included ORR per RECIST v1.1 by investigator assessment, progression-free survival (PFS), overall survival (OS), and DOR.

Median follow-up duration was 13 months at the time of analysis and in the 28 patients with measurable disease at baseline, ORR was 25% with 3 (11%) CRs and 4 (14%) PRs per central review. At the time of analysis, median DOR had not been reached and the 12-month PFS rate was 19%. ORR in patients with tumors positive for PD-L1 expression as assessed with the clinical trial assay was 38%. Pembrolizumab was very well tolerated and grade 3–4 TRAEs occurred in 5 (15%) of patients [9,10].

Based on the promising activity of pembrolizumab in refractory UC patients, the landmark phase III KEYNOTE-045 trial investigated pembrolizumab in patients with locally advanced, metastatic, or unresectable UC who had progressed on platinum-based chemotherapy or had recurrence after 12 months of chemotherapy [11].

In this study, 542 patients were randomized to pembrolizumab at a flat dosing of 200 mg IV every three weeks for two years versus chemotherapy consisting of either paclitaxel (175 mg/m^2^), docetaxel (75 mg/m^2^), or vinflunine (320 mg/m^2^) every three weeks for two years.

The primary endpoints were OS and PFS in the entire population and those with a combined positive score ≥10% for PD-L1 expression. This score consisted of the percentage of PD-L1–positive tumor cells and infiltrating immune cells relative to the total number of tumor cells from tumor tissue samples. In the OS analysis of patients with combined positive score ≥10%, there was a 43% reduction in the risk of death with pembrolizumab compared with standard chemotherapy (HR, 0.57; 95% CI, 0.37–0.88; *p* = 0.0048). The median OS was 8.0 months (95% CI, 5.0–12.3 months) with pembrolizumab versus 5.2 months (95% CI, 4.0–7.4 months) with chemotherapy.

A greater DOR was seen with pembrolizumab compared to that with chemotherapy; median DOR was not reached, and 68% of responders were considered likely to maintain a response for ≥12 months. On the contrary, the median DOR in the chemotherapy arm was 4.3 months with an estimated 35% likely to maintain a response for ≥12 months. Fewer AEs were seen with pembrolizumab compared to chemotherapy for any grade (60.9% vs. 90.2%) and for grade 3–5 AEs (15.0% vs. 49.4%).

TRAEs were lower with pembrolizumab compared to chemotherapy; fatigue (13.9% vs. 27.8%), nausea (10.9% vs. 24.3%), diarrhea (9.0% vs. 12.9%), asthenia (5.6% vs. 14.1%), and anemia (3.4% vs. 24.7%). Incidence of pruritus was higher with pembrolizumab versus with chemotherapy (19.5% vs. 2.7%). irAEs were more common in the pembrolizumab arm including thyroid abnormalities (9.4% vs. 1.6%), pneumonitis (4.1% vs. 0.4%), and colitis (2.3% vs. 0.4%).

Findings from this landmark trial represent the first immunotherapy agent to demonstrate an OS benefit over an active comparator in advanced/metastatic UC. A number of trials are investigating pembrolizumab in UC in combination with other agents for UC (Table 1).

#### 2.1.4. Avelumab

Avelumab (MSB0010718C) is a fully human IgG1 monoclonal antibody against PD-L1. In an ongoing phase I trial, avelumab is being studied in patients with metastatic or locally advanced solid tumors (NCT01772004). Patients with ECOG performance status 0-1, adequate organ function, and relapsed or refractory disease were allowed. Exclusion criteria included active CNS metastases, previous organ transplant, autoimmune disease, and cardiovascular disease. The primary outcomes are dose limiting toxicity (DLT) and confirmed best ORR by RECIST v1.1 criteria. Secondary outcomes include TRAEs, immune-related best overall response (irBOR), immune-related PFS (irPFS), PFS, OS, DOR, PD-L1 tumor expression, serum cytokines, and number of patients with anti-avelumab antibodies.

Preliminary results from metastatic UC cohort were reported recently [12]. Forty-four patients with metastatic UC unselected for PD-L1 expression received avelumab 10 mg/kg IV every two weeks until progression, unacceptable toxicity, or withdrawal. Patients had an ECOG performance status of 0-1, and received a median of 2 prior therapies. Unconfirmed ORR, PFS, and OS were evaluated. At the time of reporting of these preliminary results, patients had been followed for a median of 11 months. TRAEs occurred in 30 patients (68.2%); with infusion-related reactions, fatigue, asthenia, and nausea being most common. Grade 3 or higher TRAEs were asthenia, myositis, decreased appetite, and elevated creatinine phosphokinase (CPK)or aspartate aminotransferase (AST) (each one event). No treatment-related deaths occurred. ORR was 18.2% (8 patients; with 2 CRs and 6 PRs); stable disease was seen in 17 patients (38.6%); disease control rate was 56.8%. PD-L1 expression was evaluable in 35 patients and using 5% or higher cutoff for tumor cell staining, 12/35 (34.3%) were PD-L1+; ORR was 50.0% in PD-L1+ patients vs. 4.3% in PD-L1−patients. PFS rate at 24 weeks was 58.3% in PD-L1+ patients vs. 16.6% in PD-L1−patients. OS at 12 months was 50.9% for the overall population [12]. A randomized phase 3 trial with avelumab is ongoing (Table 1).

#### 2.1.5. Nivolumab

Nivolumab is a fully human IgG4 monoclonal antibody against PD-1 and is approved for first-line treatment in melanoma, Hodgkin lymphoma, non-small cell lung cancer, head and neck cancer, and renal cell cancer [13].

Nivolumab has been studied as monotherapy in metastatic UC in the phase I/II open-label, multicenter CheckMate 032 study [14,15]. One or more prior lines of platinum-based therapy was required for eligibility. Eligible patients were enrolled regardless of PD-L1 status and received nivolumab 3 mg/kg IV every two weeks until disease progression or discontinuation due to any reason. Primary endpoint was ORR based on RECIST v1.1 and secondary endpoints were safety, DOR, PFS, and OS [14]. Updated results were recently reported [15]. Seventy-eight patients were treated, and 65.4% had received at least two prior therapies. A confirmed investigator-assessed ORR was present in 19 (24.4%) patients. Grade 3–4 TRAEs occurred in 17 (22%) patients with elevated lipase (four (5%)) and elevated amylase (three (4%)) being most common. Fatigue, rash, dyspnea, decreased lymphocyte count, and decreased neutrophil count were also seen in two (3%) patients each. Serious AEs occurred in 36 (46%) patients of which 10% were treatment related. Two of 78 (3%) patients discontinued treatment and subsequently died because of TRAEs (grade 4 pneumonitis and thrombocytopenia) [15].

Checkmate 275 was a larger phase 2 single-arm study of 275 patients with metastatic UC who progressed on a platinum-based regimen, where nivolumab was administered at 3 mg/kg IV every two weeks until disease progression or unacceptable toxicity. Eighty-four percent patients had visceral metastases at baseline. Forty-two percent of patients had received one prior treatment while 29.3% received two or more [16]. Nivolumab showed an ORR of 19.6%; the median time to response was 1.9 months, and the median DOR had not been reached at the time of presentation of these results. Median PFS was two months (1.87 months in patients with <1% PD-L1 expression (*n* = 143) and 3.55 months in patients with PD-L1 expression of >1% (*n* = 122)). Median OS was 8.74 months overall (5.95 months in patients with PD-L1 <1% and 11.3 months in patients with PD-L1 expression of ≥1%) [16]. In a further biomarker analysis, ORR was 28.4% in patients expressing PD-L1 ≥5% and 15.8% in patients expressing PD-L1 <5%. Of note, PD-L1 expression ≥1% and ≥5% was reported for 45.9% and 30.7% of patients, respectively. Grade 3–4 TRAEs occurred in 18% of patients with fatigue and diarrhea being most common, each occurring in 2% of patients. There was one death each due to cardiovascular disease, pneumonitis, and acute respiratory failure. Quality of life improved from baseline and remained stable through the trial [16]. Nivolumab is being studied in several clinical trials in UC along with other novel immunotherapy agents, including ipilimumab (Table 1).

#### 2.1.6. Ipilimumab

Ipilimumab, a fully human IgG1 monoclonal antibody directed against cytotoxic T-lymphocyte associated antigen 4 (CTLA-4), is approved for the treatment of malignant melanoma [17]. Ipilimumab monotherapy was tested as neoadjuvant therapy in a small cohort of 12 patients with surgically resectable, T1-T2 UC with primary endpoints of safety and immune monitoring [18]. Two ipilimumab doses were evaluated; 3mg/kg and 10mg/kg. Four patients (33.3%) developed grade 3 AEs but only two (16.7%) had to delay therapy secondary to AEs, both in the 10 mg/kg arm. Eight patients (66.7%) were down-staged post-cystectomy and four patients (33.3%) with urine malignant cells by cytology or fluorescent in situ hybridization had negative urine studies post-ipilimumab [18].

#### 2.1.7. Ipilimumab and Gemcitabine-Cisplatin Combination

A multi-center phase II study combined ipilimumab with standard chemotherapy, gemcitabine and cisplatin (GC) [19]. Patients with metastatic UC received two cycles of GC alone followed by four cycles of GC with ipilimumab (GCIpi). The primary endpoint was percentage of patients alive at one year. Secondary endpoints included safety, ORR, and PFS. Thirty-six patients were enrolled; 58% had visceral metastases and 20% had liver metastases. The observed mean OS of 14.6 months was not different from that of historical controls, and median PFS was eight months. Grade 3–4 TRAEs were neutropenia (36%), thrombocytopenia (19%), anemia (25%), hyponatremia (31%), thromboembolism (11%), and renal insufficiency (19%). The most common grade 3–4 irAEs were colitis (6%), hypophysitis (3%), hyperthyroidism (1%), and rash (1%) [19]. While Ipilimumab has promising activity in UC, this trial was disappointing as it did not meet its primary endpoint with significant AEs. Ipilimumab is also being studied in combination with other novel immunotherapy agents for UC (Table 1). Results from ongoing and planned trials would provide more insight into efficacy and tolerability of such combinations.

#### 2.1.8. Nivolumab and Ipilimumab Combination

Data from the randomized phase I/II Checkmate 032 trial exploring different doses of ipilimumab with nivolumab and nivolumab monotherapy in metastatic UC were recently reported [14]. Patients who progressed after platinum-based chemotherapy received either nivolumab alone or one of two doses of nivolumab with ipilimumab. Patients either received ipilimumab 3 mg/kg with nivolumab 1 mg/kg (Nivo1/Ipi3) or ipilimumab 1 mg/kg and nivolumab 3mg/kg (Nivo3/Ipi1) given every three weeks for four cycles. This was followed by nivolumab given every two weeks until disease progression or unacceptable toxicity [14]. Median follow up was 15.2 months for the nivolumab group, 16.7 months for the Nivo3/Ipi1 group, and 7.8 months for the Nivo1/Ipi3 group. Patients receiving the combination Nivo1/Ipi3 had the highest ORR of 38.5% with 4% CRs. Combination of Nivo3/Ipi1 yielded a 26% ORR with 3% CR. Nivolumab monotherapy showed 24.4% ORR with 6% CR. While the results with Nivo1/Ipi3 were encouraging, the follow-up duration on this arm is short and ongoing analysis of activity and safety with a longer follow-up would help confirm these findings. The Nivo1/Ipi3 combination is being tested in a randomized phase 3 trial against chemotherapy in metastatic UC.

## 3. First-Line Immunotherapy in Cisplatin-Ineligible Metastatic Urothelial Carcinoma

While cisplatin-based chemotherapy is a standard first-line therapy for metastatic UC known to improve OS, up to 50% patients are not eligible to receive cisplatin due to age or other co-morbidities. Immunotherapy is a viable and promising option for this patient population, for whom outcomes have remained poor. The available results with atezolizumab and pembrolizumab in this setting are discussed below.

### 3.1. Atezolizumab

In cohort 1 of the IMvigor210 study, atezolizumab was studied as a first-line treatment in this group of chemotherapy-naïve cisplatin-ineligible patients, defined as those with a glomerular filtration rate (GFR) > 30 but <60 mL/min, hearing impairment, grade 2 or higher peripheral neuropathy, or ECOG performance status of ≥2 [20]. Atezolizumab was administered at 1200 mg given IV every three weeks until disease progression. PD-L1 on tumor infiltrating immune cells was assessed centrally as in cohort 2 of IMvigor210 study [5]. The primary endpoint was confirmed ORR assessed per RECIST v1.1. One hundred nineteen patients were treated. The median age was 73 years with 21% patients being 80 years or higher; 10% patients had received radiation, and 66% patients had visceral metastases. A majority of patients (71%) had CrCl < 60 mL/min; 13% had hearing loss of 25 dB or greater, 6% had grade 2 or higher peripheral neuropathy, and 20% patients had an ECOG PS of ≥2. Significant ORR was seen at 19%, with 5% CR, and responses were seen regardless of PD-L1 status. Median follow-up was 8.5 months and median DOR was not reached at time of data analysis. Atezolizumab was very well tolerated in this population; TRAEs were seen in 64% patients, while grade 3–4 TRAEs were only seen in 12% patients. The most common were fatigue, pruritis, and diarrhea. One grade 5 TRAE of sepsis was observed. Treatment naïve cisplatin-ineligible patients are a challenging patient population to treat with historically limited treatment options and poor tolerability of treatment. Atezolizumab appears to be a relatively well-tolerated and an efficacious agent for a subset of this population [20].

### 3.2. Pembrolizumab

Pembrolizumab has also been studied in metastatic/locally advanced cisplatin-ineligible UC patients in the phase II Keynote-052 trial. Three hundred seventy-four patients have been enrolled and early results from the first 100 patients was reported in abstract form. The overall ORR at a median follow-up of eight months was 24% and the median time to response was two months; patients with higher PD-L1 expression (>10%) had a higher ORR of 37%. The median DOR has not been reached in these 100 patients. Mature data is awaited from this study [21].

## 4. Novel Tumor-Targeted Immunotherapeutics

### 4.1. ALT-801: Tumor-Targeted Interleukin-2 Immunotherapeutic

ALT-801 is an innovative immunotherapeutic fusion protein consisting of interleukin-2 (IL-2), an approved cytokine for treatment of metastatic melanoma and renal cell carcinoma, linked to a single-chain T-cell receptor domain [22]. ALT-801 has been shown to exhibit more potent immunostimulatory and antitumor activities against solid and hematological malignancies than IL-2 alone [23]. ALT-801 was studied with GC in a phase I/II study in advanced/metastatic refractory UC [24]. Patients received GC along with ALT-801 (0.06 mg/kg/dose, d3, 5, 8, 12) on a 21-day schedule for three cycles. The initial ALT-801 dose escalation and expansion study included chemo-naïve or refractory subjects (group 1). Others were split into a two-armed phase II dose expansion study of only chemo-refractory subjects receiving either 0.06 mg/kg ALT-801 and GC or 0.06 mg/kg ALT-801 and G depending on renal function (group 2). Of 62 patients treated, 34 were chemo-refractory receiving ALT-801 and GC (group 1, *n* = 17; group 2, *n* = 17), and 76% of patients had visceral metastases. ORR was 35% (three CR, nine PR, six stable disease), median OS was 12.3 months for group 1. Grade 3–4 toxicities were significant and seen in 50% patients with the most common being thrombocytopenia (62%), neutropenia (48%), anemia (39%), lymphopenia (26%), and hypophosphatemia (19%) [24]. Combination of ALT-801 with GC resulted in significant toxicities, but there may be a therapeutic potential for combining ALT-801 with novel immunotherapies going forward.

### 4.2. ALT-803: Interleukin-15 Superagonist Complex

Interleukin-15 (IL-15) is a key factor for the development, proliferation, and activation of effector natural killer (NK) cells and CD8+ memory T cells, and ALT-803 is a novel IL-15 mutant (N72D) with enhanced IL-15 biological activity [22]. ALT-803 demonstrated durable anti-tumor activity in various solid and hematological tumor models. [25] Intravesical ALT-803 and BCG treatment reduces tumor burden bladder cancer animal models [26]. ALT-801 is also being studied with checkpoint inhibitors and chemotherapy in solid tumors. There are planned trials with ALT-801 and anti-PD-1/PD-L1 agents in advanced/metastatic UC.

## 5. Immunotherapy in Non-Muscle Invasive Bladder Cancer

With the significant activity of immunotherapy agents in advanced/metastatic UC of bladder, exploring these therapies in non-muscle invasive bladder cancer is a rational next step as after traditional intravesical BCG, around 40% patients recur and many develop muscle-invasive or metastatic disease. Ongoing trials with checkpoint inhibitors and other novel agents in BCG refractory patients would provide data in this setting (Table 2). In addition, ALT-801 is being combined with BCG in non-muscle invasive BC in an ongoing phase 1 trial (Table 2).

## 6. Immunotherapy in Adjuvant and Neoadjuvant Setting

There is no current standard of care or guidelines on use of adjuvant chemotherapy in UC. Ongoing trials with checkpoint inhibitors like atezolizumab, pembrolizumab, and nivolumab will provide important information on utility of these agents (Table 3).

While neoadjuvant chemotherapy with MVAC (methotrexate, vincristine, adriamycin, cisplatin) or GC is the standard of care in muscle-invasive BC, only a minority of patients achieve CR and a majority of patients still relapse after radical cystectomy [27]. In addition, for patients who are cisplatin-ineligible, there is no standard of care for neoadjuvant therapy. Immunotherapy agents are being utilized in several ongoing and planned trials in this setting (Table 3).

## 7. Immunotherapy and Radiation in Bladder Cancer

In an attempt to improve outcomes with radiation in localized BC, immunotherapy is being combined with radiation in an ongoing trial with pembrolizumab. Pembrolizumab in Muscle Invasive/Metastatic Bladder Cancer (PLUMMB) is an ongoing phase I trial to investigate the safety, tolerability, and effectiveness of pembrolizumab with radiotherapy. In addition, patients whose disease has metastasized will also be enrolled. Pembrolizumab will be continued after conclusion of radiation therapy for a year or until disease progression (Table 3).

## 8. Conclusions

The recent developments with immunotherapy in metastatic UC have and are further going to result in dramatic shifts in the treatment paradigm of urothelial carcinoma after almost 30 years of hiatus in this disease. Although atezolizumab is the first immunotherapy agent approved in refractory metastatic UC, several others have received FDA breakthrough therapy designation. There has been an inundation of clinical trials investigating the role of immunotherapy in metastatic, neoadjuvant, adjuvant, and non-muscle invasive UC. This rapidly changing landscape represents the most exciting era we have seen in UC. While PD-L1 expression seems to correlate to greater treatment response, the current approval of atezolizumab does not require PD-L1 testing. Many other immunotherapy trials are enriched for PD-L1 positive patients based on initial results from small patient cohorts. Ongoing and planned clinical trials should help understand the optimal sequence, combination, and duration of immunotherapy treatments in UC in various settings. The use of immunotherapy is starting to percolate to earlier stages of UC, and it remains to be seen if those who recur or progress after receiving checkpoint inhibitors would benefit from a re-challenge with a similar or a different class of immunotherapeutic agents. Although these questions remain to be answered, we have indeed come far in the management of UC and the wide array of novel immunotherapy agents has brought hope to patients with UC across the world.

## Figures and Tables

**Table 1:** Ongoing immunotherapy trials in advanced/metastatic urothelial cancer.

**Immunotherapy Trials in Metastatic or Unresectable Bladder Cancer, 1st Line Therapy Setting**				
**Intervention**	**Indication**	**Arms/Phase**	**Primary Endpoint**	**Trial ID**
Durvalumab ± Tremelimumab	Metastatic BC	chemotherapy/III	PFS	NCT02516241
*n* = 525				
Nivolumab	Metastatic BC or metastatic melanoma	±Ipilimumab/II	ORR	NCT02553642
*n* = 120				
Cisplatin/gemcitabine + Ipilimumab	BC	Single/II	1-year OS	NCT01524991
*n* = 3				
ALT-801 + cisplatin/gemcitabine	BC	Single/Ib-II	Safety	NCT01326871
*n* = 90				
CVA21 + Pembrolizumab + standard chemotherapy	BC	Single/I	Safety	NCT02043665
*n* = 90				
**Immunotherapy Trials in Metastatic or Unresectable Bladder Cancer, 2nd Line Therapy Setting**				
**Intervention**	**Indication**	**Arms/Phase**	**Primary Endpoint**	**Trial ID**
Atezolizumab	Metastatic BC	Single/IV	n/a	NCT02589717
*n* = n/a				
Atezolizumab	Metastatic BC	Taxane or Vinflunine/III	OS	NCT02302807
*n* = 931				
Avelumab	Maintenance after chemotherapy	BSC/III	OS	NCT02603432
*n* = 668				
Pembrolizumab	Metastatic BC	Taxane or Vinflunine/III	OS and PFS	NCT02256436
*n* = 470				
Atezolizumab	Metastatic BC	Single/II	ORR	NCT02108652
*n* = 439				
Pembrolizumab	Metastatic BC	Single/II	ORR	NCT02335424
*n* = 350				
Nivolumab	Metastatic BC	Single/II	ORR	NCT02387996
*n* = 242				
Pembrolizumab	Maintenance after chemotherapy	Placebo/II	PFS	NCT02500121
*n* = 200				
Tremelimumab, followed by Durvalumab vs. combo	Many cancer types	Two arms/II	ORR	NCT02527434
*n* = 76				
aCP-196+ Pembrolizumab	Metastatic BC	Single/II	ORR	NCT02351739
*n* = 74				
EphB4-HAS + Pembrolizumab	Metastatic BC	Single/II	OS	NCT02717156
*n* = 64				
Nivolumab	Multiple solid tumors	±ipilimumab/ Ib-II	ORR	NCT01928394
*n* = 1100				
PLX3397 + Pembrolizumab	Multiple solid tumors	Single/Ib-II	Safety	NCT02452424
*n* = 400				
Urelumab + Nivolumab	Metastatic BC	Single/Ib-II	Safety	NCT02253992
*n* = 200				
Ulocuplumab + Nivolumab	Multiple solid tumors	Single/Ib-II	Safety	NCT02472977
*n* = 195				
Lenvatinib + Pembrolizumab	Metastatic BC	Single/Ib-II	Safety, ORR	NCT02501096
*n* = 150				
Varlilumab + Atezolizumab	Metastatic BC	Single/Ib-II	Safety, ORR	NCT02543645
*n* = 55				
Pembrolizumab + Vorinostat	Metastatic BC or RCC	Single/Ib-II	Safety	NCT02619253
*n* = 42				
CDX-1401 + Poly ICLC + Pembrolizumab	Metastatic BC	Single/Ib-II	Safety	NCT02661100
*n* = 26				
Durvalumab ± AZD4547, Olaparib, AZD1775	Metastatic BC	Four arms/Ib	Safety	NCT02546661
*n* = 40				
Avelumab	Multiple solid tumors	Single/I	Safety	NCT01772004
*n* = 1670				
CPI-444 ± Atezolizumab	Multiple solid tumors	Single/I	Safety	NCT02655822
*n* = 534				
Lirilumab + Nivolumab	Many cancer types	Single/I	Safety	NCT01714739
*n* = 162				
Enoblituzumab (MGA271)	Many cancer types	Single/I	Safety	NCT01391143
*n* = 151				
MGD009	Many cancer types	Single/I	Safety	NCT02628535
*n* = 114				
Ramucirumab + Pembrolizumab	Multiple solid tumors	Single/I	Safety	NCT02443324
*n* = 92				
Ipililumumab + MGA271	Many cancer types	Single/I	Safety	NCT02381314
*n* = 84				
Cabozantinib + Nivolumab	Metastatic BC	±ipilimumab/I	Safety	NCT02496208
*n* = 66				
Pembrolizumab + gemcitabine or Docetaxel	Metastatic BC	Docetaxel or gemcitabine/I	Safety	NCT02437370
*n* = 38				
Enadenotucirev + Pembrolizumab	Multiple solid tumors	Single/I	Safety	NCT02636036
*n* = 30				
Interferon Gamma + Nivolumab	Metastatic BC	Single/I	Safety	NCT02614456
*n* = 15				
p53MVA + Pembrolizumab	Metastatic BC	Single/I	Safety	NCT02432963
*n* = 12				

Abbreviations: BC = bladder cancer, OS = overall survival, PFS = progression free survival, ORR = objective response rate, BSC = best supportive care, RCC = renal cell carcinoma.

**Table 2:** Ongoing Immunotherapy trials in Non-Muscle Invasive Bladder Cancer.

Immunotherapy Trials in Non-Muscle Invasive Bladder Cancer Setting				
Intervention	Indication	Arms/Phase	Primary Endpoint	Trial ID
Recombinant Adenovirus CG0070	Superficial BC after BCG failure	Single arm/III	Durable CR	NCT02365818
*n* = 122				
Pembrolizumab	Superficial BC after BCG failure	Single arm/II	CR, DFS	NCT02625961
*n* = 260				
Recombinant Adenovirus CG0070	Superficial BC after BCG failure	Four arms/II	Durable CR, CR	NCT01438112
*n* = 222				
Pembrolizumab + gemcitabine + radiation + TURBT	Superficial BC with bladder preservation	Single/II	Two-year DFS	NCT02621151
*n* = 54				
Atezolizumab	Superficial BC after BCG failure; neoadjuvant therapy for invasive BC ineligible for platinum	Single arm/II	CR	NCT02451423
*n* = 42				
Pembrolizumab + cisplatin + radiation	Superficial BC after TURBT with bladder preservation	Single/II	Safety	NCT02662062
*n* = 30				
ALT-803 + BCG	Superficial BC	BCG/Ib-II	Safety	NCT02138734
*n* = 115				
HS-410 ± BCG	Superficial BC after BCG failure	±BCG/I-II	Safety, one-year DFS	NCT02010203
*n* = 110				
ALT-801 + gemcitabine	Superficial BC after BCG failure	Single arm/Ib-II	Safety	NCT01625260
*n* = 52				
Pembrolizumab + BCG	Superficial BC after BCG failure	Single arm/I	Safety	NCT02324582
*n* = 15				

TURBT: Transuretheral Resection of Bladder Tumor; DFS: Disease Free Survival.

**Table 3:** Ongoing immunotherapy trials in neoadjuvant and adjuvant settings and with radiation in bladder cancer.

**Immunotherapy Trials in Neoadjuvant Therapy Setting**				
**Intervention**	**Indication**	**Arms/Phase**	**Primary Endpoint**	**Trial ID**
Pembrolizumab + Cystectomy	Invasive BC	Single/II	CR	NCT02736266
*n* = 90				
Atezolizumab	Invasive BC	Single/II	CR	NCT02662309
*n* = 85				
Pembrolizumab + cisplatin + gemcitabine	Invasive BC	Single/II	Downstaging	NCT02690558
*n* = 39				
Pembrolizumab + cisplatin ± gemcitabine	Invasive BC, Italian locations only	Single/Ib-II	Safety, CR	NCT02365766
*n* = 81				
**Immunotherapy Trials in Adjuvant Therapy Setting**				
**Intervention**	**Indication**	**Arms/Phase**	**Primary Endpoint**	**Trial ID**
Nivolumab	Invasive BC	Placebo/III	ORR	NCT02632409
*n* = 640				
Atezolizumab	Invasive BC, no neoadjuvant therapy, PD-L1 positive stain	Observation/III	DFS	NCT02450331
*n* = 440				
**Combination of Immunotherapy and Radiation Therapy in Superficial Urothelial Carcinoma**				
Pembrolizumab + radiation	Superficial BC, ineligible for surgery or concurrent chemotherapy	Single/I	Safety	NCT02560636
*n* = 34				

BC = bladder cancer, ORR = objective response rate.
